# Technology for Triple Fortification of Salt with Folic Acid, Iron, and Iodine

**DOI:** 10.1111/1750-3841.14730

**Published:** 2019-08-08

**Authors:** Oluwasegun Modupe, Kiruba Krishnaswamy, Levente L. Diosady

**Affiliations:** ^1^ Dept. of Chemical Engineering and Applied Chemistry Univ. of Toronto 200 College Street Toronto Ontario Canada M5S 3E5; ^2^ Dept. of Biomedical, Biological and Chemical Engineering & Dept. of Food Science Univ. of Missouri 44 Agricultural Engineering Building Columbia MO 65211, USA

**Keywords:** fortification, micronutrients, folic acid, anemia, spina bifida

## Abstract

**Abstract:**

As many of the maternal and child health complications result from folic acid, iron, and iodine deficiencies; it makes sense to combat these simultaneously. We have developed cost‐effective technology to deliver these three micronutrients simultaneously through salt. Our goal was to retain at least 70% of the micronutrients during 6 months of storage. The fortified salt was formulated by spraying a solution that contained 2% iodine and 0.5% or 1% folic acid onto salt and adding encapsulated ferrous fumarate. The formulated triple fortified salt contained 1,000 ppm iron, 50 ppm iodine, and 12.5 or 25 ppm folic acid. The spray solution and the salt were stored for 2 and 6 months respectively at 25, 35, and 45 °C 60 to 70% relative humidity. Even at 45 °C, over 70% of both iodine and folic acid were retained in the salt. The best formulation based on the color of the salt and stability of iodine and folic acid contained 12.5 ppm folic acid, 50 ppm iodine, and 1,000 ppm iron. These results indicate that iron, iodine, and folic acid can be simultaneously delivered to a vulnerable population through salt using the technology described. Also, the quality control of the process can be developed around pteroic acid that was detected as a primary degradation product of folic acid.

**Practical Application:**

The technology developed is already transferred to India for industrial scale up. When fully operational, the technology will simultaneously solve iron, iodine, and folic acid deficiencies in vulnerable populations at a very low cost.

## Introduction

The consequences of micronutrient deficiencies remain an important global health issue (Allen, De Benoist, Dary, & Hurrell, 2006; Müller & Krawinkel, 2005). The contribution of micronutrient deficiencies to the global disease burden is significant, as it is a risk factor in many adverse clinical conditions(Black, 2003). For instance, iron deficiency affects about one‐third of the world population. It leads to millions of maternal and infant deaths annually (McLean, Cogswell, Egli, Wojdyla, & De Benoist, 2009). Other micronutrients with high‐deficiency prevalence include iodine, vitamin A, and zinc (Müller & Krawinkel, 2005).

Micronutrient deficiencies significantly impact human health. Iron deficiency can cause anemia and has adverse effects on working capacity, motor, and mental development (Allen, 2000). Folic acid deficiency is a risk factor for neural tube defects, stroke, and cardiovascular disease (Crider, Bailey, & Berry, 2011; de Benoist, 2008). The effect of iodine deficiency is tied to its adverse impact on thyroid hormone, a hormone that regulates cellular metabolism, early growth, and development. Some of the consequences of iodine deficiencies are goiter, mental retardation, impaired growth, and neurological disorders (Kapil, 2007).

The coexistence of two or more micronutrient deficiencies can aggravate their clinical consequences in humans. Iron deficiency anemia along with megaloblastic anemia caused by lack of folic acid leads to excessive loss of red blood cells and can be lethal (Batata, Spray, Bolton, Higgins, & Wollner, 1967). Also, there are cases where the absorption and metabolism of two or more micronutrients are interdependent (Christakos, Dhawan, Porta, Mady, & Seth, 2011). In such cases, the lack of one micronutrient may significantly affect the absorption and/or metabolic function of other micronutrient(s) (Teucher, Olivares, & Cori, 2004). For instance, vitamin C enhances the absorption of iron, and the metabolism of folic acid and vitamin B_12_ is intertwined (Lynch & Cook, 1980; Shane & Stokstad, 1985). Therefore, there is a need to address some multiple micronutrient deficiencies concurrently.

Although no age group or sex is free from the bane of micronutrient deficiencies, the effect is prominent in pregnant women and children (Bailey, West Jr, & Black, 2015; Lopez, Cacoub, Macdougall, & Peyrin‐Biroulet, 2016). The effect of micronutrient deficiencies on pregnant women is serious as it can directly impact on the fetus which may lead to birth defects (Gazzali et al., 2016). The Food Engineering Group at the University of Toronto developed salt fortified with iron and iodine (DFS) for the purpose of solving the problem of iron and iodine deficiencies, which are key factors to maternal health (Yao O. Li, Diosady, & Wesley, 2010). Salt is the vehicle of choice for the fortificant because it is an absolute necessity in the diet. Also, salt is consumed at a uniform rate in each population; hence, an adequate, and safe amount of micronutrients can be added to salt to meet the needs of the target population. In India, an adult consumes about 10 g of salt per day (Powles et al., 2013); based on this, the target concentrations of iron and iodine in the salt were 1,000 and 50 ppm, respectively. It was estimated that this will deliver 10 and 0.5 mg of iron and iodine respectively daily to a person per day. Iron is added in the form of a microencapsulated solid premix; the encapsulation provides a physical barrier between iron and iodine that is sprayed on salt in solution form (Yao Olive Li, 2009; Yao O. Li et al., 2010; Yadava, Olive Li, Diosady, & Wesley, 2012). The industrial production of DFS is now reaching about 50 million households in India (L. Diosady, Mannar, & Menon, 2018).

Hence, the objective of this study was to evaluate the feasibility of the process developed to simultaneously deliver at least 30% of RDA of iodine, iron, and folic acid through salt by studying the stability of folic acid and iodine in salt over time under controlled conditions of temperature and humidity, expected in the target geography. The stability of iron was not considered because iron cannot be degraded under the set conditions (L. L. Diosady, Alberti, Ramcharan, & Venkatesh Mannar, 2002; Yao Olive Li, Yadava, Lo, Diosady, & Wesley, 2011). The goal is to have more than 70% of the added micronutrients retained in salt after 6 months of storage. The impact of light and moisture content was not considered because in a parallel study, it was shown that light does not impact the stability of folic acid and iodine, and the premix is coated with fat‐based polymer to prevent the impact of moisture in the salt. Additionally, the study elucidated the chemistry of interaction among the micronutrients by determining the products of degradation of the fortificants; a step towards developing a robust quality control system for the technology.

## Materials and Experimental Methods

### Materials

Refined salt (∼400 µm diameter) was obtained from *Sifto Canada Corp*. Potassium iodate was obtained from Sigma‐Aldrich Chem. Folic acid was obtained from Bulk Pharm. Inc. Ferrous fumarate was obtained from Dr. Paul‐Lohmann Chem. Sodium carbonate and potassium iodide, used for the analysis of folic acid and iodine respectively, were obtained from Caledon Lab Chem. Sulfuric acid, and starch indicator, used for the analysis of iodine were obtained from EMD and Lab Chem Inc., respectively. Soy stearin, hydroxypropyl methyl cellulose (HPMC), semolina, Crisco shortening and titanium (IV) oxide, used in the production of iron premix, were obtained from JVS Food Ltd., Dow Chem. Company, Unico Inc., J.M. Smucker Co., and ACROS Organics, respectively. Iron premix was obtained from JVS Food Ltd. All chemicals used for the fortification of salt were food grade whereas those used for analysis were ACS grade.

### Experimental methods

#### Formulation and storage of spray solution

Iodine and folic acid were added as an aqueous solution sprayed onto salt. The spray solutions were prepared using reverse osmosis purified water. The first sets of spray solutions were prepared using carbonate buffer at pH 9. The carbonate buffer was prepared using sodium carbonate and sodium bicarbonate. The spray solution contained 1% to 2% (w/v) folic acid and 1% to 3% (w/v) iodine at pH 9 achieved by adding 0.1 to 0.3 M sodium carbonate (Table 1). The impact of the carbonate solution on the solubility of folic acid was evaluated.

**Table 1:** Formulation design for the preliminary spray solution

Spray solutions	1	2	3
Buffered solution concentration (M)	0.1	0.2	0.3
Iodine (w/v, %)	1	2	3
Folic acid (w/v, %)	1	2	1.8

Based on the results obtained from an initial study, to reduce the impact of yellow color of folic acid, and to simplify the procedure a second set of spray solutions containing 0.5% to 1% (w/v) folic acid and 3.37% (w/v) potassium iodate (equivalent of 2% [w/v] iodine) were prepared with 0.1 M sodium carbonate solution (Table 2). Subsequently, the concentration of iodine in the solution (2% [w/v]) was fixed consistently with industrial practice in India. To study the effect of pH on the stability of the micronutrients in solution, sodium carbonate solution was used to adjust the pH of some of the solutions to 9 and others to 11.2. Sodium citrate (1% [w/v]) was added to some of the spray solutions to study the impact of citrate on the stability of folic acid. The exact amount of sodium carbonate to be added to obtain pH 9 was evaluated. The stability of the micronutrients in the spray solution was monitored for 2 months at 25, 35, 45 °C and 70% relative humidity.

**Table 2:** Formulation design for the final spray solution used for making triple fortified salt

Spray solutions	Folic acid (%)	Iodine (%)	Citrate (%)
pH adjusted to 9			
1	0.5	0	0
2	1.0	0	0
3	0	2	0
4	0.5	2	0
5	1.0	2	0
6	0.5	0	1
7	1.0	0	1
8	1.0	2	1
9	0.5	2	1
pH adjusted to 11.2			
10	0.5	0	0
11	1.0	0	0
12	0.5	2	0
13	1.0	2	0

#### Production of iron premix

The process described by Li, Diosady, and Wesley (2009) and Yadava et al. (2012) was modified for iron premix processing at the last stage of encapsulation. Four premix combinations were used for making fortified salt (Table 3). The color masked (30% TiO_2_) extrudate was first coated with 5% (w/w) hydroxypropyl methylcellulose (HPMC) via a fluidized bed unit operation, then with 5% (w/w) soy stearine (SS) via pan coating (Premix B). Premix C was coated with 10% (w/w) SS whereas Premix D was coated with 10% HPMC. Premix A encapsulated with 5% HPMC and 5% soy stearin was obtained from our industrial partner, JVS Foods Pty, Jaipur, India. The final size of the premix particles was 300 to 400 µm; this matches the size of salt particles, hence prevent segregation from the salt given that its density is very close to that of salt. These iron premixes were then blended with salt.

**Table 3:** Design of the coating of the premix

Premix samples	HPMC (%)	Soy stearin (%)
A^a^ [IN (5% HPMC + 5% SS)]	5	5
B [Lab (5% HPMC + 5% SS)]	5	5
C [IN (10% SS)]	0	10
D [IN (10% HPMC)]	10	0

a Premix sample was obtained from the pilot plant. SS, soy stearin; HPMC, hydroxypropyl methylcellulose. The samples are described in Figure 5. footnote.

#### Formulation of fortified salt

**Figure 1:**
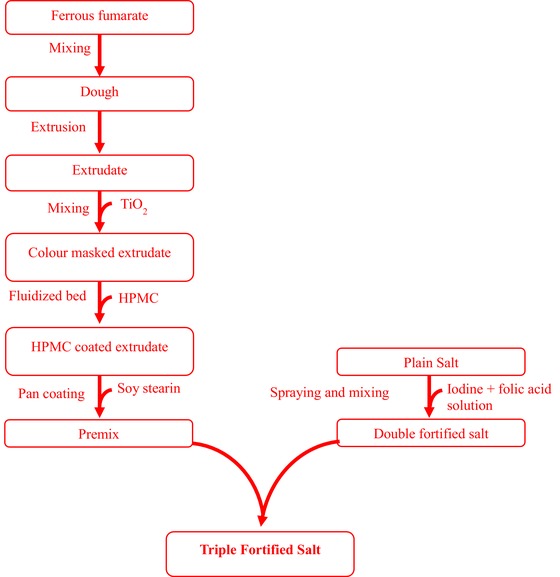
Flowchart for the triple fortification of salt. HPMC, hydroxypropyl methylcellulose; TiO_2_, titanium dioxide.

Figure 1 illustrates all the steps involved in the formulation of the triple fortified salt (TFS). Refined salt (2 kg), obtained from *Sifto Canada Corporation*, was added into the ribbon blender and agitated until a uniform granular powder was obtained. A freshly prepared spray solution (5 mL) containing 2% iodine and/or 0.5% to 1% folic acid was sprayed on the salt. This was thoroughly mixed in the ribbon blender. The salt fortified with iodine and/or folic acid was dried at room temperature overnight. The fortified salt was returned to the ribbon blender and the iron premix (Premix A, B, C, or D) was added and gently mixed to prevent the premix coating rubbing off the extrudate. The resultant salts contained 12.5 ppm folic acid;

12.5 ppm folic acid and 50 ppm iodine;
25 ppm folic acid and 50 ppm iodine;
12.5 ppm folic acid and 1,000 ppm iron;
12.5 ppm folic acid, 50 ppm iodine and 1,000 ppm iron; and
25 ppm folic acid, 50 ppm iodine and 1,000 ppm iron, respectively.

The salt samples were divided by a salt splitter glassware into three portions, each salt set was stored at ambient conditions (25 °C), 35 °C 60 to 70%RH and 45 °C 60 to 70%RH. The stability of folic acid and iodine in the salt was monitored over a period of 6 months.

#### Folic acid analysis

For the spray solution, samples were diluted with 0.1 M Na_2_CO_3_ at ratio 1:1,000 (v/v) and the absorbance of the resulting solution was measured at 256 nm. For the salt samples, 5 g of the samples were dissolved in 0.1 M Na_2_CO_3_ (10 mL) in a falcon tube. The solution was mixed using a vortex mixer for 2 min and filtered with a 0.45 µm syringe filter. The absorbance of the filtrate was immediately read at 256 nm using Varian Cary 50 Spectrophotometer. At this wavelength, the absorbance of the potential folic acid degradation products is insignificant.

#### Iodine analysis

Method 33.149, described by the Association of Official Analytical Chemists (AOAC), was used for iodine quantification in salt and spray solutions (Association of Official Analytical Chemists, 1984). In this method, iodate is reduced to iodine and titrated with sodium thiosulfate using a starch indicator.

#### Color analysis

The *L* ^*^ *a* ^*^ *b* ^*^ color properties of salt samples were determined by colorimeter (Chroma Meter CR‐400/40, Konica Minolta Photo Imaging U.S.A., Inc., Mahwah, NJ).

#### Elucidating the chemistry of interaction among the fortificants

The products of folic acid degradation were determined over a period of 6 months. Using ultra‐high‐performance liquid chromatography‐mass spectrometry, the total ion chromatogram of a freshly prepared TFS and after storage for 6 months was determined and subjected to differential analysis using Compound Discoverer Software to detect what new compound has been formed in the stored salt. The software was directed to identify compounds whose concentrations in the salt after storage for 6 months was at least 200% higher than their concentrations when the salt was freshly prepared; these were considered to be products of folic acid degradation. One of the potential products of degradation of folic acid was subjected to molecular analysis with Chem3D 17.1 Software to estimate some of its partial atomic charges, bond length, and angle in order to predict its molecular structure. The structure of pteroic acid was obtained from the ChemACX database attached to the software. The Huckel charges on the atoms of pteroic acid were estimated with a sub menu under the calculation menu of the software whereas the bond length and angles were estimated with sub menus under the structure menu.

#### Statistical analysis

The results are expressed as a mean of four replicates ± SD. The data were subjected to 1‐way ANOVA using SPSS Software and the differences between means were considered significant at *P* < 0.05.

## Results and Discussion

### Preliminary studies: increase in concentration of micronutrients in the spray solution

A spray solution containing 0.35% (w/v) of iodine and folic acid had been prepared for the formulation of salt containing folic acid and iodine (McGee, Sangakkara, & Diosady, 2017). In the preliminary study, the concentration of folic acid and iodine was increased to 1% and 2% and 1% and 3%, respectively. High moisture content is one of the factors that accelerates iodine loss in iodized salt (Allen et al., 2006). Excess moisture added while spraying may weaken the coat on the iron premix in the resultant salt, which allows for interaction of iron and iodine. Increase in concentration of micronutrients in the spray solution, results in less spray solution to be evaporated from salt. This will ultimately minimize the loss of iodine in the fortified salt.

**Figure 2:**
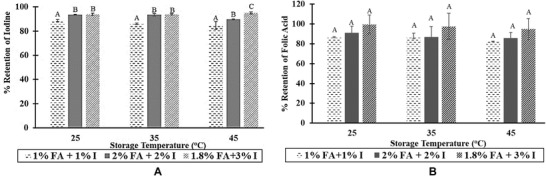
Stability of Iodine (A) and folic acid (B) in spray solution (2‐month preliminary study). FA, folic acid; I, iodine.

Stability studies carried out on the spray solution showed that both iodine and folic acid were relatively stable in the solution. In all the spray solutions, 80% to 95% of the added micronutrients were retained after 2 months of study (Figure 2). While increasing the concentration of the micronutrients led to significant increase in the stability of iodine, stability of folic acid was not significantly impacted by the increased concentration.

The solubility of folic acid improved with higher concentration of sodium carbonate. However, in a solution containing more than 1% (w/v) folic acid, the solubility of the micronutrients in the solution dropped with time. This can be a significant problem in small salt plants where spray solutions can be stored for a month.

### Stability of iodine and folic acid in the newly formulated spray solution for triple fortification of salt

Given the solubility problem encountered with the spray solution prepared in the preliminary study, new sets of spray solutions were formulated. The concentration of folic acid was maintained at 1% (w/v), and the possibility of reducing the concentration to 0.5% (w/v) while still meeting 50% RDA of folic acid in fortified salts was investigated. To be consistent with industrial practice, the concentration of iodine was maintained at 2% in the spray solution. Also, the previous method used for spray solution formulation was simplified. The carbonate buffer was replaced with sodium carbonate solution. This reduced the number of steps required for making the spray solution. To obtain pH 9, 0.742 g sodium carbonate, 3.37 g potassium iodate, and 1 g folic acid were dissolved in 100 mL of water. In all the samples 70% to 100% of folic acid and iodine were retained after 2 months of storage.

#### Impact of pH on the stability of micronutrients

**Figure 3:**
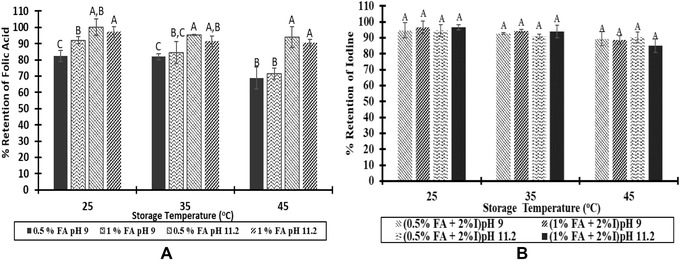
Effect of pH on folic acid (A) and iodine (B) stability in the spray solutions (2‐month storage). FA, folic acid; I, iodine.

The impact of pH was significant on the stability of folic acid in the spray solution. For 0.5% folic acid, as pH decreased, the percentage retention of folic acid decreased. For 1% folic acid, as pH decreased, the percentage retention of folic acid decreased only at 45 °C storage temperature (Figure 3A). For iodine, neither a change in folic acid concentration nor a change in pH affected the iodine stability. Although, a higher pH is favored for the stability of folic acid, a higher pH may not be desirable if addition of vitamin B_12_, a micronutrient that is essential in metabolism of folic acid, is contemplated.

#### Effect of folic acid concentration on the stability of iodine and folic acid in spray solutions

**Figure 4:**
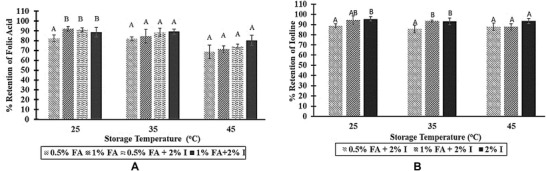
Effect of folic acid concentration on folic acid (A) and Iodine (B) stability in the spray solution (2‐month storage). FA, folic acid; I, iodine.

Folic acid concentration in the spray solution did not significantly affect the stability of folic acid and iodine in the spray solutions, except at 25 °C (Figure 4A and B), the percentage of folic acid retained in the spray solution A (containing 0.5% folic acid) was significantly reduced compared to other solutions (Figure 4B). This trend was also observed in the earlier spray solutions; however, we do not understand what is responsible for the trend. Iodine seems to have stabilized folic acid in some of the solutions. The same trend was reported by McGee et al. (2017).

Color is one of the factors that affects the acceptability of fortified food (Mejia, 1994). The reduction of folic acid concentration in the solution has a high potential for improving the color of salt and maintaining stability of both I and FA, while still meeting the target concentration of folic acid in salt. Moreover, the reduction may reduce the increased cost of salt fortification.

#### Effect of citrate on the stability of iodine and folic acid in spray solutions

Folic acid is susceptible to degradation by oxidizing and reducing agents (Fennema, 1996). Hence, we investigated citrate as a folic acid stabilizer. Citrate did not significantly improve the stability of folic acid in the spray solution; it only slightly improved the stability of folic acid at 45 °C and 60% to 70% RH. This confirmed that citrate is not needed in the spray solution formulation, reducing the potential impact of its cost on the overall cost of making TFS. Given the results from the stability study carried out on the spray solution and future opportunity to accommodate vitamin B_12_ into the spray solution, the optimal spray solution for triple fortification of salt consisted of 0.5% to 1% folic acid and 2% iodine at pH 9.

### Stability of iodine and folic acid in TFS after 6 months

**Figure 5:**
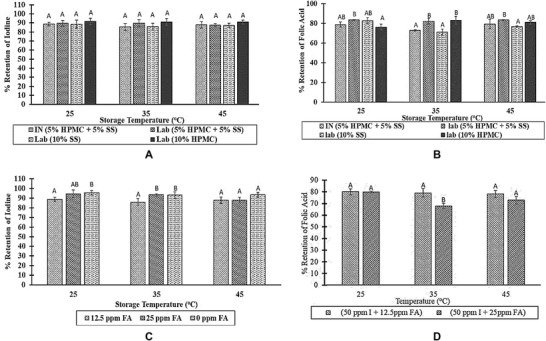
The effect of extrudate coat on stability of iodine (A) and folic acid (B), and the effect of folic acid concentration on stability of iodine (C) and folic acid (D) in triple fortified salt (6‐month storage). The result is presented as a percentage of folic acid (12.5 or 25 ppm) and iodine (50 ppm) initially added to salt. All of the salt samples in a and b contained 1,000 ppm iron and a percentage of 12.5 ppm folic acid, the initial concentration whereas those in c and d contained 1,000 ppm iron and a percentage 50 ppm iodine, the initial concentration. Premix A is IN (5% HPMC + 5% SS), premix B is Lab (5% HPMC + 5% SS), premix C is Lab (10% SS), and premix D is Lab (10% HPMC). Two of the premix samples (A and C) had perfect coat whereas premix B and D had some grey spots. FA, folic acid; I, iodine; HPMC, hydroxypropyl methylcellulose; SS, soy stearin.

Salt stays on the distribution channels for an average of 2 months (L. Diosady, Yusufali, Oshinowo, & Laleye, 2006). Also, the target population buys small amount of salt that is typically consumed within 2 months. Hence, the goal is to have at least 70% of the micronutrients retained in the fortified salt after 6 months. After 6 months of storage, 70% to 85% of folic acid and 85% to 95% of iodine were retained in all the samples, even at 45 °C and 60% to 70% RH (Figure 5). This confirmed that the technology can be used to deliver iron, iodine, and folic acid simultaneously through salt. Given the established distribution channel of salt, TFS has the potential of reaching millions of vulnerable households that otherwise may not have access to diets with sufficient iron, iodine, and folic acid.

Although, the impact of the coating material on the stability of folic acid in the TFS did not follow any trend, TFS prepared with imperfectly coated premix samples retained marginally more folic acid. This trend was most noticeable in samples stored at 35 °C (Figure 5B). Iron may be stabilizing folic acid in the TFS samples. This trend had been observed in rice fortified with iron and folic acid (Yao O. Li, Diosady, & Jankowski, 2011). The apparent imperfection of the iron premix did not impact on the stability of iodine in the salt (Figure 5A). Folic acid may be playing a stabilizing role in the salt; it may have shielded iodine from the effect of the exposed iron of the premix. Of the three coating combinations, the 5% HPMC and 5% soy stearin resulted in the most rapid sinking of premix and salt particles in water. This is desirable because floating premix is likely to be to be washed away during initial washing of the salt in the household.

In the tested range, folic acid concentration (12.5–25 ppm) did not significantly affect the stability of folic acid in TFS except at 35 °C (Figure 5D). At this temperature, the retention of folic acid in 12.5 ppm folic acid salt was significantly higher than that in 25 ppm folic acid salt. Also, lowering the concentration of folic acid in the salt [from 25 ppm described by McGee et al. (2017) to 12.5 ppm] improved the color of TFS (Table 4). The reduction in folic acid concentration will have the tendency of positively impacting the acceptance of the salt. Unlike folic acid, the stability of iodine was impacted by folic acid concentration; the effect was significant at lower temperatures. The percentage of iodine retained in the salt containing 12.5 ppm folic acid was significantly lower at 25 and 35 °C. However, in all cases, more than 85% of iodine added was retained after 6 months of storage of the salt samples (Figure 5C). Hence, the impact of folic acid concentration on iodine stability in TFS may be negligible when weighed against the improved color of TFS at 12.5 ppm. Moreover, the salt fortified 12.5 ppm folic acid can still deliver the target concentration of folic acid (50% RDA of folate).

**Table 4:** *L* ^*^ *a* ^*^ *b* ^*^ color properties of salt samples

Salt sample	*L* ^*^	a^*^	*b* ^*^
Iron + I	98.74	−0.02	1.15
Iron + I +12.5 ppm folic acid	95.43	−1.53	8.43
Iron + I +25 ppm folic acid	94.27	−2.92	13.50

The concentrations of iron and iodine are 1,000 and 50 ppm respectively in all the salt samples. *L* ^*^ (+ = lighter; ‐ = darker); *a* ^*^ (+ = red; ‐ = green); *b* ^*^ (+ = yellow; − = blue).

The results showed that technology developed for triple fortification can be used to deliver folic acid, iron, and iodine at a very low cost. It is estimated that the addition of the micronutrients will add less than 0.20 USD per person in a year as an addition to the cost of buying salt. This implies that the most vulnerable population affected by these micronutrient deficiencies can likely be reached with this technology.

### Understanding the chemistry of interaction among micronutrients in TFS

The loss of iodine from TFS is through a redox reaction between ferrous iron and iodate that leads to production of elemental iodine from potassium iodate (Diosady et al., 2002). The iodine is then lost via sublimation. Because iron and iodine have been physically separated by the microencapsulation of the extruded ferrous, most of the iodine is retained even at high temperature and humidity (45 °C and 70% RH; Figure 5A). However, there is a less well‐defined knowledge of how folic acid is lost in the salt. The three molecules that make up folic acid can be dissociated; the amide group on C2 can also be dissociated. Folic acid is relatively stable at high temperatures; in solid state, it starts to degrade at 180 °C with the cleavage of folic acid to glutamic acid and pteroic acid. At 200 °C, it degrades to 2‐amino‐6‐methyl‐1H‐pteridin‐4‐one (6‐methyl pterin), 4‐ aminobenzoic acid, and glutamic acid (Gazzali et al., 2016). Nagaraja, Vasantha, and Yathirajan (2002) indicated that oxidative cleavage of folic acid yields the same products as thermal degradation. The same authors also described the products of reductive cleavage as p‐amino‐benzoyl glutamic acid and 6‐methyl pterin. Thermal degradation of folic acid is very unlikely in this study as the maximum storage temperature was 45 °C. However, redox cleavage of folic acid is a possible pathway for the degradation.

Identifying the products of degradation of micronutrients is a feasible route for elucidating the chemistry of the interaction among the micronutrients. The differential analysis of the spectra obtained from freshly prepared solution of folic acid and iodine and from the same solution stored for some period reliably provided information on the products of degradation of folic acid and iodine. The TIC spectra of freshly prepared TFS and after its storage for 6 months were obtained and subjected to differential analysis using the Compound Discoverer Software.

**Figure 6:**
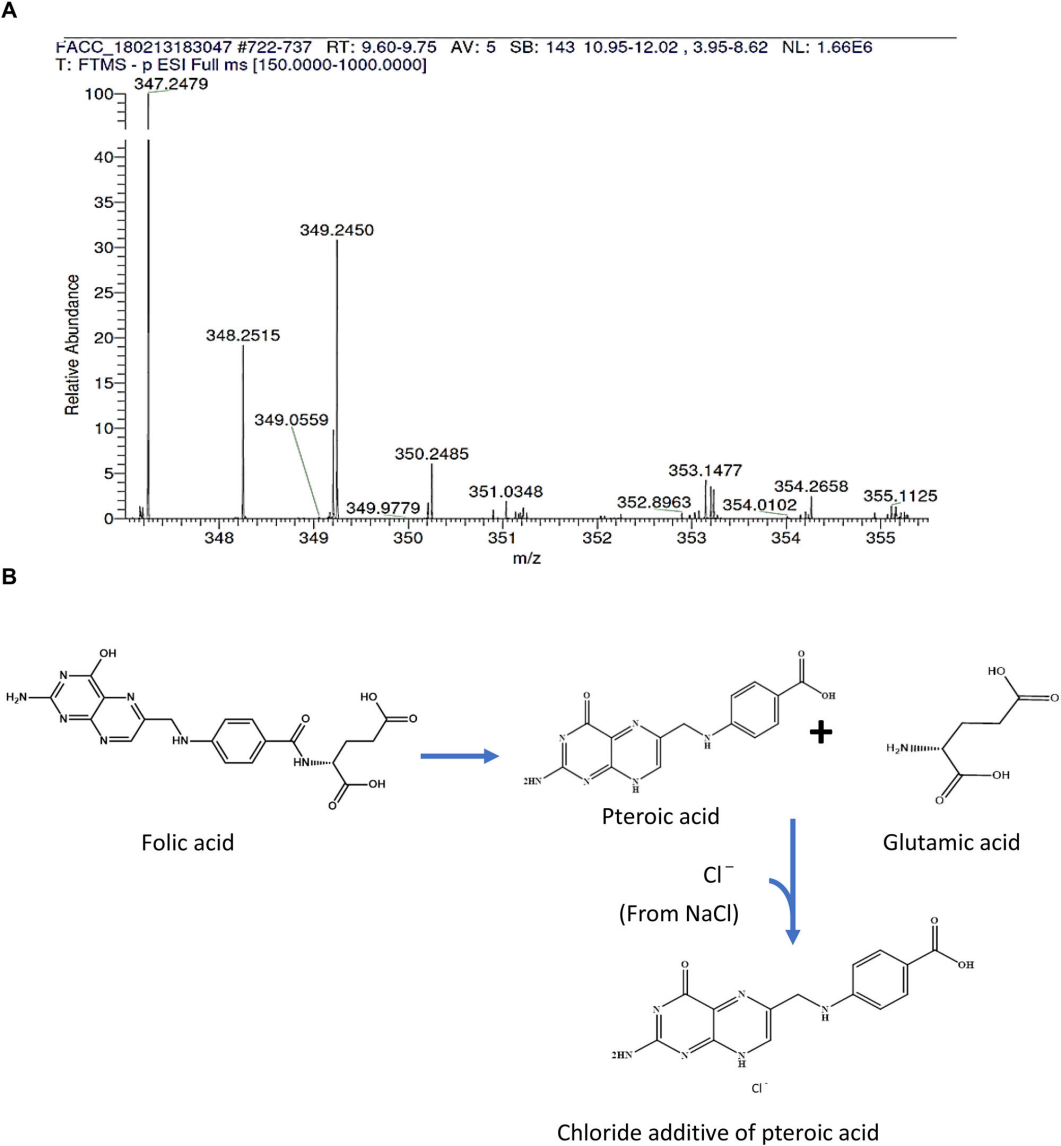
(A) The UHPLC‐MS TIC spectra of the compound detected (chloride additive of pteroic acid). (B) Possible pathway for the formation of chloride additive of pteroic acid.

The differential analysis on the TIC spectra only found one compound (348 g/mol) to be a valid product of degradation of folic acid. The fingerprint of spectra of the product showed that it is a chloride compound (Figure 6A). The subtraction of the atomic mass of chlorine (35.5) from the molecular weight of the compound (348 g/mol) suggested that the compound detected is a chloride additive of pteroic acid. The possible pathway for the formation of this product is illustrated in Figure 6B. Given that pteroic acid is detected as a product of folic acid degradation, we believe that oxidative degradation is the likely mechanism for folic acid degradation in salt. The stability of folic acid in the salt fortified with an iron premix with imperfect coating supports this hypothesis by retaining marginally more folic acid. The Fe^2+^, being a reducing agent, likely slowed the oxidation in the salt system.

Pteroic acid is made up of two of the three moieties of folic acid (6‐methyl pterin and amino benzoic acid). The third moiety (glutamic acid) was not detected. This may imply that other products of degradation of folic acid were not ionized because of the solvent used (acetonitrile solution, pH 6.5). Also, it may imply that the concentration of the products of degradation was below the detection limit of the analytical method. The former happened to be the case; glutamic acid was detected when formic acid with a lower pH was used as the solvent. The elucidation of this reaction has broadened our understanding of the degradation of folic acid in the salt. The analytical method can be used to detect the degradative products of folic acid in salt as a measure towards having a robust quality control for the process of triple fortification of salt.

Chem3D is a software that is used to view and analyze the 3D structure of chemical compounds; its Gaussian component allows for prediction of molecular structure, chemical, and energy properties of chemical compounds. Using the Software, the Huckel charges on the atoms of pteroic acid and the bond lengths and angles between the atoms were estimated. N8 has the maximum positive partial charge (0.50) of the possible atoms that is available for chlorine interaction. Also, the bond length of C5–N8 and N8–C12, and the bond angle of C5–N8–C12 favors N8 as the atom likely to interact with a chlorine atom.

## Conclusions

Given that at least 70% of the of iodine and folic acid were retained in the TFS after 6 months of storage, the technology developed can simultaneously deliver folic acid, iron, and iodine to vulnerable populations. The best formulation has 12.5 ppm folic acid, 50 ppm iodine, and 1,000 ppm iron, which will deliver at least 50% of the RDA of the micronutrients based on a daily consumption of 10 g salt. The insight into the products of degradation of folic acid can be leveraged in developing a robust quality control procedure for triple fortification of salt. Although the color of the salt was improved by reducing the concentration of folic acid, co‐encapsulation of folic acid and iron will be the way forward in dealing with the color issue of the TFS.

## Funding

This research is funded by the Saving Lives at Birth consortium through Grand Challenges Canada.

## Authors’ Contributions

Oluwasegun Modupe and Kiruba Krishnaswamy carried out the experiments. Oluwasegun Modupe designed the experiment and prepared this manuscript. Diosady Levente was the Principal Investigator who supervised the study and edited the manuscript.

## Conflicts of Interest

The authors have no conflicts of interest.
